# Osmotic Induction of Angiogenic Growth Factor Expression in Human Retinal Pigment Epithelial Cells

**DOI:** 10.1371/journal.pone.0147312

**Published:** 2016-01-22

**Authors:** Moritz Veltmann, Margrit Hollborn, Andreas Reichenbach, Peter Wiedemann, Leon Kohen, Andreas Bringmann

**Affiliations:** 1 Department of Ophthalmology and Eye Hospital, University of Leipzig, Leipzig, Germany; 2 Paul Flechsig Institute of Brain Research, University of Leipzig, Leipzig, Germany; 3 Helios Klinikum Aue, Aue, Germany; National Centre for Scientific Research, 'Demokritos', GREECE

## Abstract

**Background:**

Although systemic hypertension is a risk factor of age-related macular degeneration, antihypertensive medications do not affect the risk of the disease. One condition that induces hypertension is high intake of dietary salt resulting in increased blood osmolarity. In order to prove the assumption that, in addition to hypertension, high osmolarity may aggravate neovascular retinal diseases, we determined the effect of extracellular hyperosmolarity on the expression of angiogenic cytokines in cultured human retinal pigment epithelial (RPE) cells.

**Methodology/Principal Findings:**

Hyperosmolarity was induced by the addition of 100 mM NaCl or sucrose to the culture medium. Hypoxia and oxidative stress were induced by the addition of the hypoxia mimetic CoCl_2_ and H_2_O_2_, respectively. Alterations in gene expression were determined with real-time RT-PCR. Secretion of bFGF was evaluated by ELISA. Cell viability was determined by trypan blue exclusion. Nuclear factor of activated T cell 5 (NFAT5) expression was knocked down with siRNA. Hyperosmolarity induced transcriptional activation of bFGF, HB-EGF, and VEGF genes, while the expression of other cytokines such as EGF, PDGF-A, TGF-β1, HGF, and PEDF was not or moderately altered. Hypoxia induced increased expression of the HB-EGF, EGF, PDGF-A, TGF-β1, and VEGF genes, but not of the bFGF gene. Oxidative stress induced gene expression of HB-EGF, but not of bFGF. The hyperosmotic expression of the bFGF gene was dependent on the activation of p38α/β MAPK, JNK, PI3K, and the transcriptional activity of NFAT5. The hyperosmotic expression of the HB-EGF gene was dependent on the activation of p38α/β MAPK, ERK1/2, and JNK. The hyperosmotic expression of bFGF, HB-EGF, and VEGF genes was reduced by inhibitors of TGF-β1 superfamily activin receptor-like kinase receptors and the FGF receptor kinase, respectively. Hyperosmolarity induced secretion of bFGF that was reduced by inhibition of autocrine/paracrine TGF-β1 signaling and by NFAT5 siRNA, respectively. Hyperosmolarity decreased the viability of the cells; this effect was not altered by exogenous bFGF and HB-EGF. Various vegetable polyphenols (luteolin, quercetin, apigenin) inhibited the hyperosmotic expression of bFGF, HB-EGF, and NFAT5 genes.

**Conclusion:**

Hyperosmolarity induces transcription of bFGF and HB-EGF genes, and secretion of bFGF from RPE cells. This is in part mediated by autocrine/paracrine TGF-β1 and FGF signaling. It is suggested that high intake of dietary salt resulting in osmotic stress may aggravate neovascular retinal diseases via stimulation of the production of angiogenic factors in RPE cells, independent of hypertension.

## Introduction

Age-related macular degeneration (AMD) is the main cause of visual impairment and blindness in people aged over 65 years in developed countries [1]. The wet form of AMD is characterized by the development of choroidal neovascularization and subretinal edema resulting from dysfunction of the retinal pigment epithelium (RPE), outer retinal hypoxia, and abnormalities in Bruch's membrane [2]. Dysfunction of the RPE and retinal edema result in a progressive decrease of the visual acuity due to photoreceptor degeneration [3]. Vascular endothelial growth factor (VEGF) is the most relevant hypoxia-induced angiogenic factor that promotes choroidal neovascularization and edema [4]. RPE cells are an important source of VEGF in the retina [5]. The role of VEGF in pathological neovascularization has provided evidence for the use of anti-VEGF agents as treatment of choroidal neovascularization [6,7]. However, in more than half of patients anti-VEGF therapy does not improve the visual acuity, and about 10% of the patients do not respond to the treatment [8]. In addition, anti-VEGF agents may induce activation of a compensatory angiogenic signaling [9].

In the last years, it became evident that increased production of VEGF by RPE cells alone is not sufficient to promote choroidal neovascularization [10]. The finding that the synergistic action of other proangiogenic factors is required for the angiogenic effect of VEGF [11,12] has led to the suggestion that future treatments of wet AMD should include inhibition of further factors to obtain a greater benefit regarding antiangiogenesis [7]. Such angiogenic factors that are produced by the RPE are, for example, platelet-derived growth factor (PDGF), basic fibroblast growth factor (bFGF), and heparin-binding epidermal growth factor-like growth factor (HB-EGF) [13–15]. Intraocular bFGF has been shown to induce experimental choroidal neovascularization [16]. bFGF and VEGF act synergistically on retinal vascular endothelial cells [17]. The effect of bFGF is in part mediated by stimulation of VEGF secretion [18,19]. HB-EGF is upregulated in the retina in proliferative retinopathies and after ischemia-reperfusion [20,21]. It has various protective effects on retinal cells such as inhibition of osmotic glial cell swelling [21] and protection against light-induced photoreceptor degeneration [22], a pathogenic aspect of AMD. HB-EGF stimulates the proliferation and migration of RPE cells, as well as the production of VEGF [14].

In addition to advanced age, race, genetic markers, sun light exposure, smoking, and nutritional factors, systemic hypertension is a risk factor of wet AMD [23–25]. The major condition that causes acute hypertension is the increase in the blood osmolarity following high intake of dietary salt (NaCl) [26,27]. The blood pressure-raising effect of dietary salt increases with age [28]. However, although hypertension is a risk factor of wet AMD, antihypertensive medications do not affect the risk of early AMD and even increase the risk of wet AMD [23,29]. This may suggest that, in addition to hypertension, high osmolarity may aggravate neovascular retinal diseases. Hyperosmotic stress has various effects in the retina and RPE including a decrease of the standing potential of the eye [30] that originates from the RPE [31], alterations in the membrane potential and resistance of the RPE [32], and opening of the outer blood-retinal barrier constituted by the RPE [33]. We found recently that hyperosmotic stress induces the production of VEGF in RPE cells [34]. However, it is not known whether the expression of further growth factors and cytokines, that are involved in the pathogenesis of neovascular and proliferative retinal diseases, is osmotically regulated in RPE cells. Therefore, we compared the alterations in the gene expression of different cytokines induced by hyperosmotic stress and found that, in addition to the VEGF gene, bFGF and HB-EGF genes are transcriptionally activated in RPE cells in response to extracellular hyperosmolarity. Because outer retinal hypoxia is a condition which contributes to the development of AMD [2], we compared the hyperosmotic and hypoxic regulation of angiogenic factor expression in RPE cells. In addition, high extracellular NaCl is known to cause oxidative stress [35,36], a pathogenic factor of AMD [37]. Therefore, we investigated whether antioxidant vegetable polyphenols may inhibit the hyperosmotic expression of bFGF and HB-EGF genes in RPE cells.

## Materials and Methods

### Ethics Statement

The study followed the tenets of Declaration of Helsinki for the use of human subjects. The use of human material was approved by the Ethics Committee of the University of Leipzig (approval #745, 07/25/2011). Tissues were obtained with the written informed consent from relatives of all donors.

### Materials

All tissue culture components and solutions were purchased from Gibco BRL (Paisley, UK). Recombinant human bFGF and recombinant human HB-EGF were purchased from R&D Systems (Abingdon, UK). AG1478, LY294002, PD98059, and SP600125 were obtained from Calbiochem (Bad Soden, Germany). SB203580 was from Tocris (Ellisville, MO). Human-specific small interfering RNA (siRNA) against nuclear factor of activated T cell 5 (NFAT5) and nontargeted control siRNA were obtained from Qiagen (Hilden, Germany). All other agents used were from Sigma-Aldrich (Taufkirchen, Germany), unless stated otherwise. The following antibodies were used: a neutralizing goat anti-bFGF (R&D Systems), a neutralizing goat anti-HB-EGF (R&D Systems), a neutralizing rabbit anti-transforming growth factor (TGF)-β (pan specific; R&D Systems), a rabbit anti-human NFAT5 (1:200; Santa Cruz Biotechnology, Dallas TX), a rabbit anti-human β-actin (1:1000; Cell Signaling, Frankfurt/M., Germany), and anti-rabbit IgG conjugated with alkaline phosphatase (1:2000; Cell Signaling).

### Cell culture

Eyes were obtained from post-mortem donors without reported eye disease within 48 h of death. RPE cells were prepared and cultured as following. After removing the vitreous and neural retina, RPE cells were mechanically harvested, separated by digestion with 0.05% trypsin and 0.02% EDTA, and washed two times with phosphate-buffered saline. The cells were suspended in complete Ham F-10 medium containing 10% fetal bovine serum, glutamax II, and gentamycin, and were cultured in tissue culture flasks (Greiner, Nürtingen, Germany) in 95% air/5% CO_2_ at 37°C. Cells of passages 2, 4, and 5 were used. Isoosmotic control medium had an extracellular osmolarity of 287.5 ± 1.6 mosm/kg H_2_O (n = 5). Hyperosmotic media were made up by adding NaCl or sucrose. Addition of 30 and 100 mM NaCl to the culture medium resulted in osmolarities of 346.9 ± 2.3 and 486.7 ± 3.3 mosm/kg H_2_O, respectively. The hypoosmotic medium (60% osmolarity) was made up by adding distilled water. Chemical hypoxia was induced by addition of CoCl_2_ (150 μM), and oxidative stress was induced by addition of H_2_O_2_ (20 μM). The cells were preincubated with the pharmacological inhibitors for 30 min.

### Real-time RT-PCR

Total RNA was extracted from RPE cells by using the RNeasy Mini Kit (Qiagen, Hilden, Germany). The quality of the RNA was analyzed by agarose gel electrophoresis. The A_260_/A_280_ ratio of optical density was measured using the NanoDrop1000 device (peQLab, Erlangen, Germany), and was between 1.95 and 2.03 for all RNA samples, indicating sufficient quality. After treatment with DNase I (Roche, Mannheim, Germany), cDNA was synthesized from 1 μg of total RNA using the RevertAid H Minus First Strand cDNA Synthesis kit (Fermentas, St. Leon-Roth, Germany).

Real-time RT-PCR was performed with the Single-Color Real-Time PCR Detection System (BioRad, Munich, Germany) using the primer pairs described in Table 1. The PCR solution contained 1 μl of cDNA, specific primer set (0.2 μM each) and 10 μl of a 2x mastermix (iQ SYBR Green Supermix; BioRad) in a final volume of 20 μl. The following conditions were used: initial denaturation and enzyme activation (one cycle at 95°C for 3 min); denaturation, amplification and quantification, 45 cycles at 95°C for 30 s, 58°C for 20 s, and 72°C for 45 s; melting curve, 55°C with the temperature gradually increased (0.5°C) up to 95°C. The amplified samples were analyzed by standard agarose gel electrophoresis. The mRNA expression was normalized to the level of ß-actin mRNA. The changes in mRNA expression were calculated according to the 2^-ΔΔCT^ method (CT, cycle threshold), with ΔCT = CT_target gene_—CT_actb_ and ΔΔCT = ΔCT_treatment_—ΔCT_control_. Real-time PCR efficiency (E) was calculated according to the equation E = 10^[-1/slope]^-1. The efficiencies for different genes were similar in the investigated range between 0.025 and 50 ng cDNA (for example, for *ACTB*, 0.95; *BFGF*, 1.03; *HBEGF*, 1.05; and *VEGFA*, 0.95).

**Table 1:** Primer pairs used in PCR experiments. s, sense. as, anti-sense.

Gene / Accession	Primer sequence (5’→3’)	Amplicon (bp)
**ACTB**	_s_ ATGGCCACGGCTGCTTCCAGC	237
NM_001101	_as_ CATGGTGGTGCCGCCAGACAG	
**VEGFA**_**188, 164, 120**_		479, 407, 275
NM_003376.5	_s_ CCTGGTGGACATCTTCCAGGAGTA	
NM_001287044.1	_as_ CTCACCGCCTCGGCTTGTCACA	
NM_001025370.2		
**BFGF**	_s_ AGAGCGACCCTCACATCAAG	234
NM_002006	_as_ ACTGCCCAGTTCGTTTCAGT	
**HBEGF**	_s_ TGCCTGTAGCTTTCCTGGTCCC	258
NM_001945	_as_ CCCCACCTCCAACCTTCTCGG	
**HGF**	_s_ AGGAGAAGGCTACAGGGGCAC	267
NM_001010932.1	_as_ TTTTTGCCATTCCCACGATAA	
**PDGFA**	_s_ CAAGACCAGGACGGTCATTT	190
NM_033023	_as_ CCTGACGTATTCCACCTTGG	
**TGFB1**	_s_ GGGACTATCCACCTGCAAGA	239
NM_000660	_as_ CCTCCTTGGCGTAGTAGTCG	
**TGFB2**	_s_ ACGTCTCAGCAATGGAGAAGA	195
NM_001135599.2	_as_ ATTCGCCTTCTGCTCTTGTTT	
**PEDF**	_s_ TGCAGGCCCAGATGAAAGGG	342
NM_002615.4	_as_ TGAACTCAGAGGTGAGGCTC	
**IL1B**	_s_ GGGCCTCAAGGAAAAGAATC	205
NM_000576	_as_ TTCTGCTTGAGAGGTGCTGA	
**EGF**	_s_ CAGGGAAGATGACCACCACT	187
NM_001963	_as_ CAGTTCCCACCACTTCAGGT	
**IL6**	_s_ TACCCCCAGGAGAAGATTCC	175
NM_000600.3	_as_ TTTTCTGCCAGTGCCTCTTT	
**IL8**	_s_ TCAGTGCATAAAGACATACTCC	198
NM_000584.3	_as_ TATGAATTCTCAGCCCTCTT	
**NFAT5**	_s_ TCACCATCATCTTCCCACCT	174
XM_005255777.1	_as_ CTGCAATAGTGCATCGCTGT	

### mRNA stability

Cells were first treated with NaCl (100 mM) or vehicle (double-distilled water) for 12 h followed by addition of actinomycin D (5 μg/ml). Total RNA was isolated 1.5, 3, 4.5, and 6 h after addition of actinomycin D, and mRNA expression was determined by real-time RT-PCR analysis.

### Cell viability

Cell viability was determined by trypan blue exclusion. Cells were seeded at 5x 10^4^ cells per well in 6-well plates. After reaching a confluency of ~90%, the cells were cultured in medium containing 0.5% fetal calf serum for 16 h and then in serum-containing iso- or hyperosmotic medium (+ 100 mM NaCl) for 24 h. Cells were preincubated with bFGF and/or HB-EGF (10 ng/ml each) for 30 min in isoosmotic medium before administration of the factors in the hyperosmotic medium. After trypsinization, the cells were stained with trypan blue (0.4%), and the number of viable (non-stained) and dead (stained) cells were determined using a hemocytometer.

### siRNA transfection

Cells were seeded at 7 x 10^4^ cells per well in 12-well culture plates and were allowed to growth up to confluency of 60–80%. Thereafter, the cells were transfected with NFAT5 siRNA and nontargeted siRNA (each 10 or 50 nM), respectively, using HiPerfect reagent (Qiagen) in F-10 medium containing 10% fetal bovine serum (Invitrogen) according to the manufacturer's instructions. After 24 h, the medium was removed and fresh medium without serum was added for 16 h. Thereafter, hyperosmotic medium (+ 100 mM NaCl) without serum was added for 6 h. Total RNA was extracted, and the mRNA levels were determined with real-time RT-PCR analysis.

### ELISA

Cells were cultured at 3 x 10^3^ cells per well in 12-well plates. At a confluency of ~90%, the cells were cultured in serum-free medium for 16 h. siRNA-transfected cells were serum-deprived for 2 h. Subsequently, the culture medium was changed, and the cells were stimulated with a hyperosmotic medium (+ 100 mM NaCl) in the absence and presence of pharmacological inhibitors. The supernatants were collected after 6 h, and the levels of bFGF and HB-EGF, respectively, in the cultured media (100 μl) were determined with ELISA (R&D Systems).

### Western blot analysis

siRNA-transfected cells were serum-deprived for 16 h and then treated with iso- and hyperosmotic media (+ 100 mM NaCl) for 6 h, respectively. Then, the media were removed, the cells were washed twice with prechilled phosphate-buffered saline (pH 7.4; Invitrogen, Paisley, UK), and the monolayers were scraped into 80 μl of lysis buffer (50 mM Tris-HCl pH 8.0, 5 mM EDTA, 150 mM NaCl, 0.5% NP-40, 1% protease inhibitor cocktail) and agitated at 4°C for 30 min. Total cell lysates were centrifuged at 13,000 x *g* for 10 min, and the supernatants were analyzed by immunoblotting. Equal amounts of protein were separated by 10% SDS-polyacrylamide gel electrophoresis; immunoreactive bands were visualized using 5-bromo-4-chloro-3-indolyl phosphate/nitro blue tetrazolium.

### Statistics

For each test, at least three independent experiments were carried out in triplicate. Data are expressed as means ± SEM. Statistical analysis was made using Prism (Graphpad Software, San Diego, CA). Significance was determined by one-way ANOVA followed by Bonferroni's multiple comparison test and Mann-Whitney *U* test, respectively, and was accepted at *P*<0.05.

## Results

### Hyperosmotic regulation of cytokine gene expression

**Fig 1:**
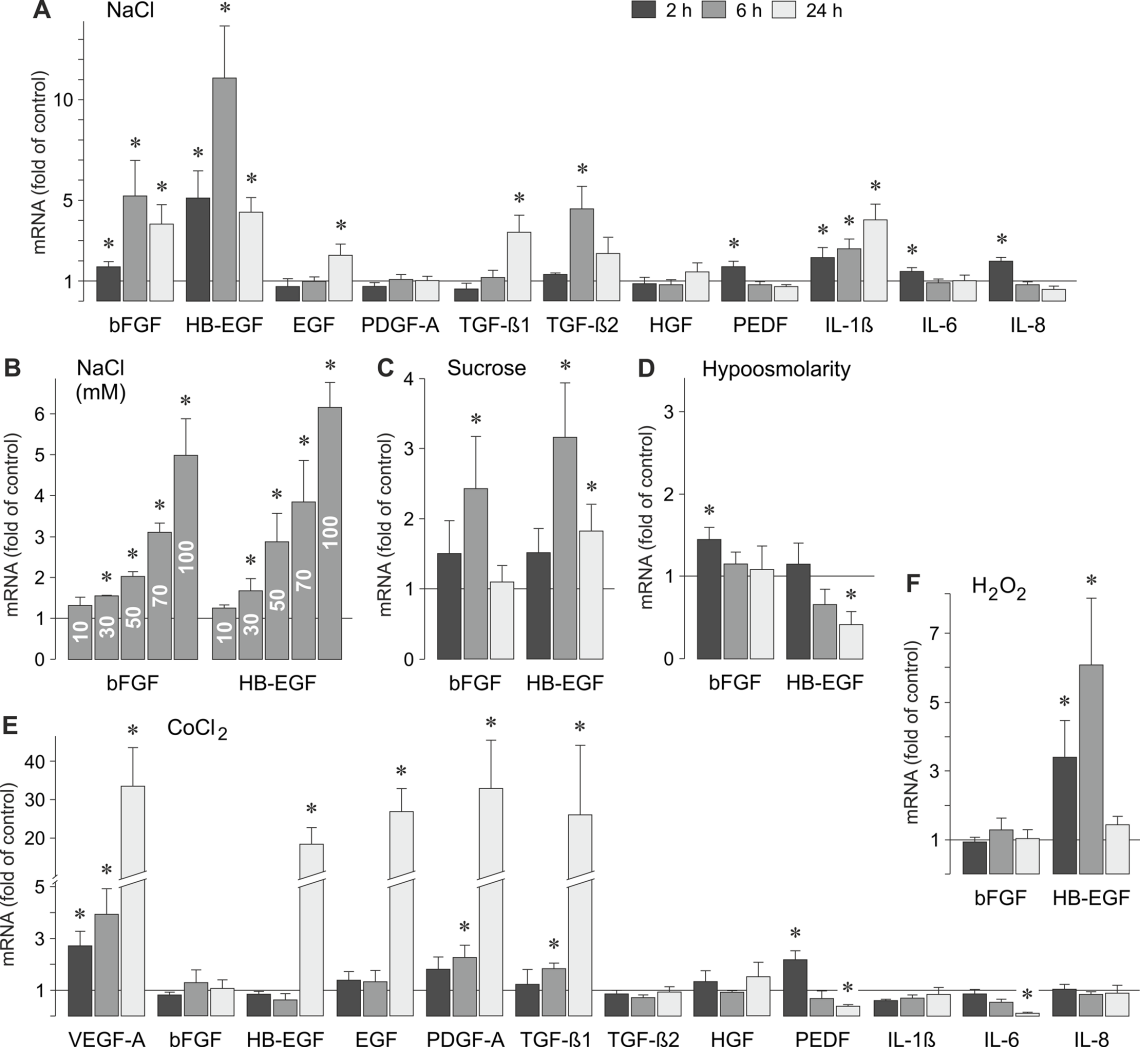
Regulation of gene expression of growth factors and cytokines in human RPE cells. The alterations in the gene expression in cells cultured 2, 6, and 24 h under different conditions were determined with real-time RT-PCR analysis. The following conditions were tested: hyperosmolarity induced by addition of NaCl (100 mM; **A,B**) or sucrose (100 mM; **C**) to the culture medium, hypoosmolarity (60% osmolarity; **D**), chemical hypoxia induced by addition of CoCl_2_ (150 μM; **E**), and oxidative stress induced by addition of H_2_O_2_ (20 μM; **F**). (**B).** Dose-dependent effect of high extracellular NaCl on the cellular levels of bFGF and HB-EGF mRNAs. The cells were cultured in media which were made up hyperosmotic by addition of 10 to 100 mM NaCl. Means ± SEM of 3–7 independent experiments using cells from different donors. Significant difference *vs*. unstimulated control: *P<0.05.

We found recently that hyperosmotic stress induces the production of VEGF in RPE cells [34]. In order to compare the alterations in the gene expression of different growth factors and cytokines induced by hyperosmotic stress, we carried out real-time RT-PCR analysis of lysates derived from cultured human RPE cells. Stimulation of the cells with a hyperosmotic medium (+ 100 mM NaCl) induced (in addition to the upregulation of the VEGF gene expression recently described [34]) time-dependent increases in the expression of bFGF, HB-EGF, TGF-β1, TGF-β2, and interleukin (IL)-1β genes (Fig 1A). No or moderate alterations were found in the expression of epidermal growth factor (EGF), PDGF-A, hepatocyte growth factor (HGF), pigment epithelium-derived factor (PEDF), IL-6, and IL-8 genes (Fig 1A). The data suggest that hyperosmotic stress induces gene expression of various angiogenic factors in RPE cells, including bFGF and HB-EGF. The effect of high extracellular NaCl on the cellular levels of bFGF and HB-EGF mRNAs was dose-dependent (Fig 1B). Increases in the levels of bFGF and HB-EGF transcripts were also found in cells cultured in medium that was made up hyperosmotic by addition of 100 mM sucrose (Fig 1C). A hypoosmotic medium (60% osmolarity) induced a small increase in the expression of the bFGF gene, and a time-dependent decrease in the expression of the HB-EGF gene (Fig 1D).

Inhibition of RNA polymerase II by actinomycin D (5 μg/ml) completely abrogated the increases in bFGF and HB-EGF mRNA levels induced by stimulation of the cells with hyperosmotic (+ 100 mM NaCl) medium for 6 and 12 h, respectively (data not shown). The stabilities of bFGF and HB-EGF mRNAs did not differ between iso- and hyperosmotic (+ 100 mM NaCl) conditions up to 6 h after addition of actinomycin D (not shown). The data suggest that the hyperosmotic increases of the bFGF and HB-EGF mRNA levels were due to stimulation of gene transcription and not due to alterations of the mRNA stability. The transcriptional inhibitor actinomycin D also decreased the levels of bFGF and HB-EGF transcripts under control conditions (not shown), suggesting that the genes of both factors are constitutively expressed in the cells.

### Hypoxic regulation of cytokine gene expression

Hypoxia is a main inducer of angiogenic factors including VEGF in RPE cells [11,38]. In order to compare hypoxia-induced alterations in the gene expression of different cytokines, we carried out real-time RT-PCR analysis of RPE cells cultured in the presence of the hypoxia mimetic CoCl_2_ [39]. As shown in Fig 1E, chemical hypoxia induced delayed gene expression of various growth factors in RPE cells including VEGF, HB-EGF, EGF, PDGF-A, and TGF-β1 genes. The levels of bFGF, TGF-β2, HGF, IL-1β. and IL-8 transcripts were not altered by chemical hypoxia. The level of IL-6 transcripts was decreased in a time-dependent manner, while the level of PEDF mRNA displayed a biphasic regulation, with up- and downregulation after 2 and 24 h of stimulation (Fig 1E). The data suggest that hyperosmolarity and hypoxia regulate differentially the gene expression of various growth factors in RPE cells. The level of HB-EGF mRNA, but not of bFGF mRNA, was also increased in response to oxidative stress induced by addition of H_2_O_2_ (Fig 1F).

### Intracellular signaling involved in hyperosmotic bFGF and HB-EGF gene expression

**Fig 2:**
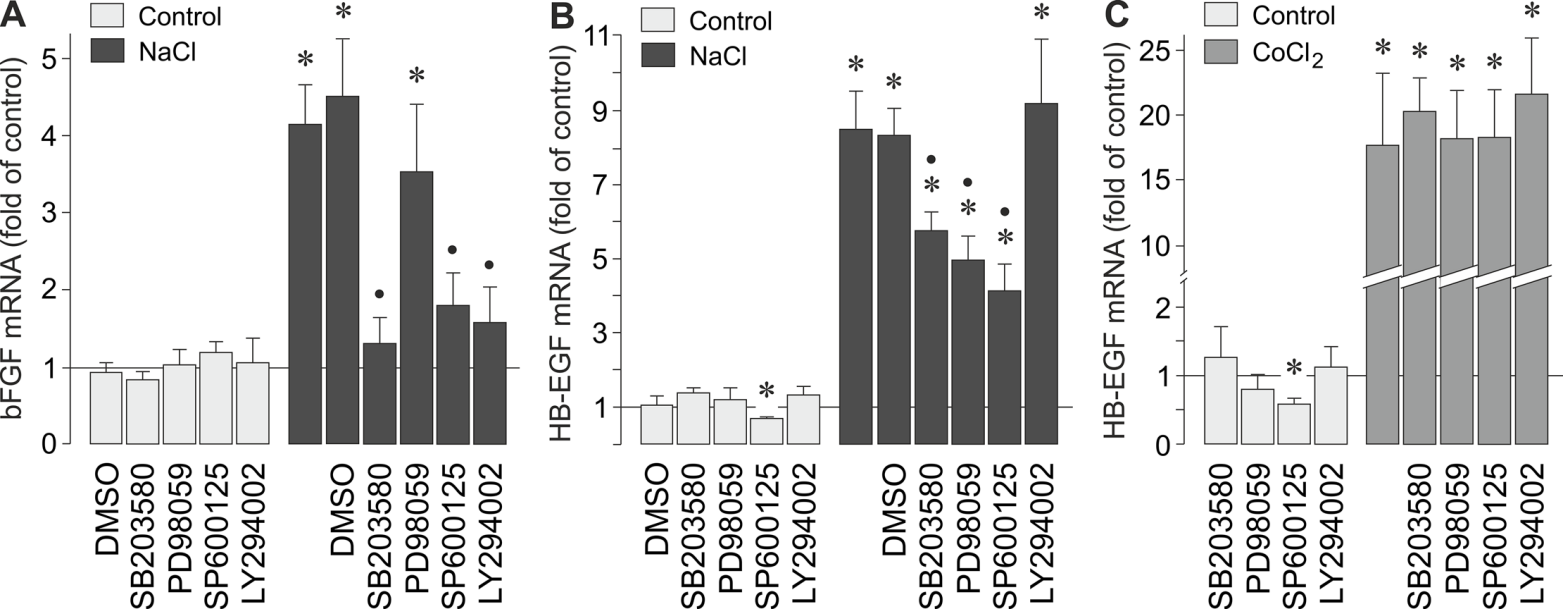
Intracellular signaling involved in osmotic and hypoxic induction of bFGF and HB-EGF gene expression. **A,B.** Cellular levels of bFGF (**A**) and HB-EGF mRNAs (**B**) were determined with real-time RT-PCR analysis in cells cultured 6 h in iso- (control) and hyperosmotic (+ 100 mM NaCl) media, respectively. (**C**). The mRNA level was determined in cells cultured 24 h under control conditions and in the presence of CoCl_2_ (150 μM), respectively. The following pharmacological inhibitors were tested: the inhibitor of p38α/β MAPK activation, SB203580 (10 μM), the inhibitor of ERK1/2 activation, PD98059 (20 μM), the JNK inhibitor SP600125 (10 μM), and the inhibitor of PI3K-related kinases, LY294002 (5 μM). The vehicle control was made with dimethylsulfoxide (DMSO; 1:1000). Means ± SEM of 3–7 independent experiments using cells from different donors. Significant difference *vs*. unstimulated control: *P<0.05. Significant difference *vs*. NaCl control: •*P*<0.05.

In order to determine the intracellular signaling that regulates the hyperosmotic expression of bFGF and HB-EGF genes, we tested pharmacological blockers of key intracellular signal transduction molecules. As shown in Fig 2A, the hyperosmotic expression of the bFGF gene was significantly (*P*<0.05) decreased by inhibitors of p38α/β mitogen-activated protein kinases (p38α/β MAPK), c-Jun NH_2_-terminal kinase (JNK), and phosphatidylinositol-3 kinase (PI3K) activation. Inhibition of extracellular signal-regulated kinases 1 and 2 (ERK1/2) activation had no significant effect (Fig 2A). The hyperosmotic expression of the HB-EGF gene was significantly (*P*<0.05) decreased by inhibitors of p38α/β MAPK, ERK1/2, and JNK signal transduction pathways, while the inhibitor of PI3K activation, LY294002, was without effect (Fig 2B). On the other hand, the CoCl_2_-induced expression of the HB-EGF gene was not significantly altered in the presence of the inhibitors tested (Fig 2C). The data suggest that various intracellular signal transduction pathways are activated in RPE cells under hyperosmotic conditions, and that the hyperosmotic expression of bFGF and HB-EGF genes is differentially regulated by distinct pathways.

### Extracellular signaling involved in hyperosmotic bFGF and HB-EGF gene expression

**Fig 3:**
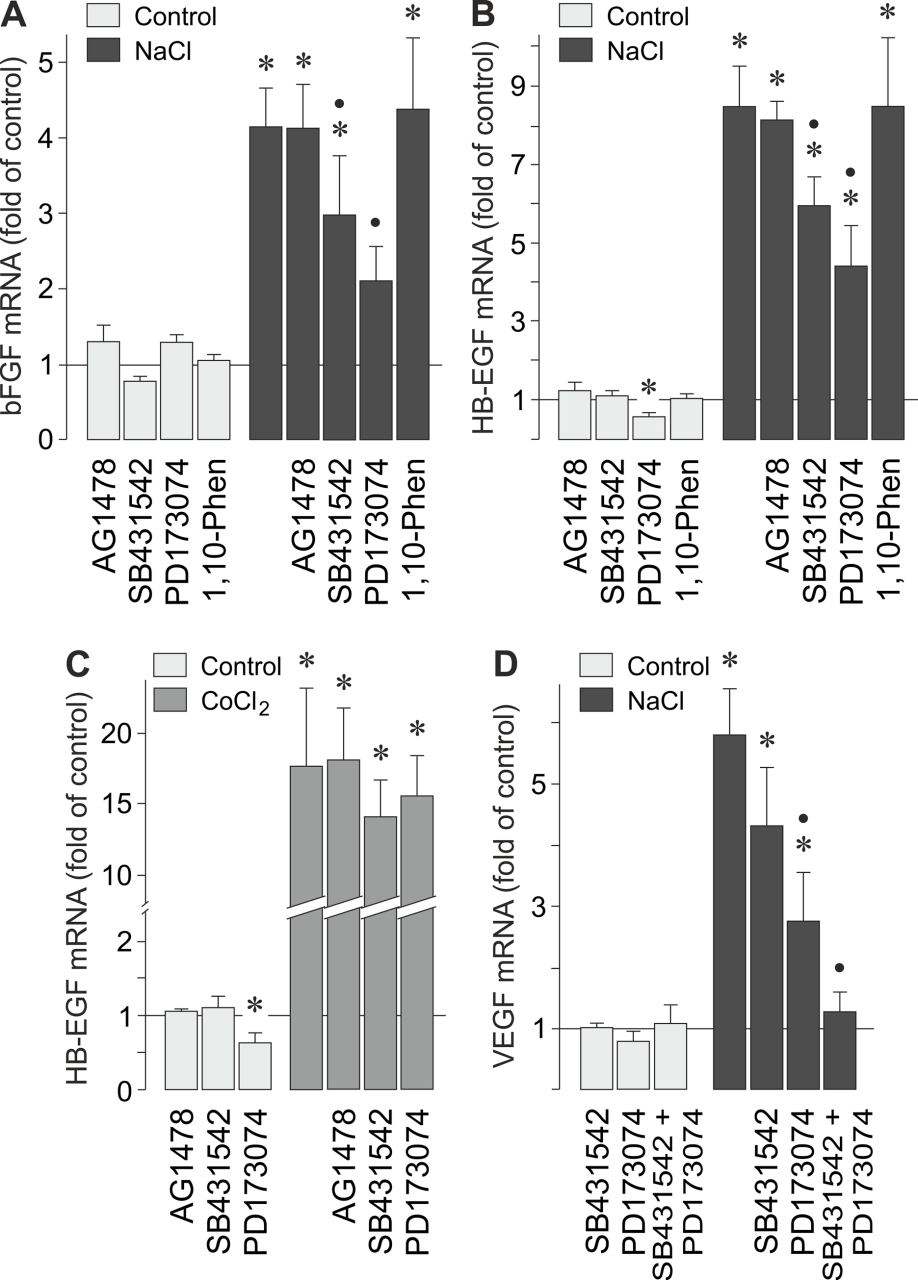
Extracellular signaling involved in the osmotic induction of bFGF, HB-EGF, and VEGF gene transcription. Cellular levels of bFGF (**A**), HB-EGF (**B**), and VEGF mRNAs (**D**) were measured with real-time RT-PCR analysis in cells cultured 6 h in iso- (control) and hyperosmotic (+ 100 mM NaCl) media. The hypoxic expression of the HB-EGF gene (**C**) was determined in cells cultured 24 h in the absence (control) and presence of CoCl_2_ (150 μM). The following pharmacological inhibitors were tested: the inhibitor of the EGF receptor tyrosine kinase, AG1478 (600 nM), the inhibitor of TGF-β1 superfamily activin receptor-like kinase receptors, SB431542 (10 μM), the FGF receptor kinase inhibitor, PD173074 (500 nM), and the broad-spectrum metalloproteinase inhibitor 1,10-phenanthroline (1,10-Phen; 10 μM). Means ± SEM of 3–7 independent experiments using cells from different donors. Significant difference *vs*. unstimulated control: *P<0.05. Significant difference *vs*. NaCl control: •*P*<0.05.

In order to determine whether autocrine/paracrine growth factor receptor signaling is required for the hyperosmotic expression of bFGF and HB-EGF genes in RPE cells, we tested inhibitors of receptor kinases. The hyperosmotic expression of bFGF (Fig 3A) and HB-EGF genes (Fig 3B) was significantly (*P*<0.05) reduced by inhibitors of TGF-β1 superfamily activin receptor-like kinase receptors and the FGF receptor kinase, SB431542 and PD173074, respectively. The hyperosmotic expression of bFGF (Fig 3A) and HB-EGF genes (Fig 3B) was not decreased by the inhibitor of the EGF receptor tyrosine kinase, AG1478, and the broad-spectrum matrix metalloproteinase inhibitor 1,10-phenanthroline. The CoCl_2_-induced expression of the HB-EGF gene was not significantly altered in the presence of the inhibitors tested (Fig 3C). These data suggest that autocrine/paracrine TGF-β1 and FGF signaling, but not EGF receptor signaling, is involved in mediating the effect of hyperosmolarity on the expression of bFGF and HB-EGF genes.

We found recently that the VEGF gene is transcriptionally activated in RPE cells under hyperosmotic conditions [34]. As shown in Fig 3D, the inhibitor of TGF-β1 superfamily activin receptor-like kinase receptors, SB431542, and the inhibitor of the FGF receptor kinase, PD173074, reduced the hyperosmotic expression of the VEGF gene. Co-administration of both inhibitors fully abrogated the hyperosmotic expression of the VEGF gene (Fig 3D). This suggests a critical role of autocrine/paracrine TGF-β1 and FGF signaling in the hyperosmotic induction of VEGF in RPE cells.

### NFAT5 induces hyperosmotic bFGF gene expression

**Fig 4:**
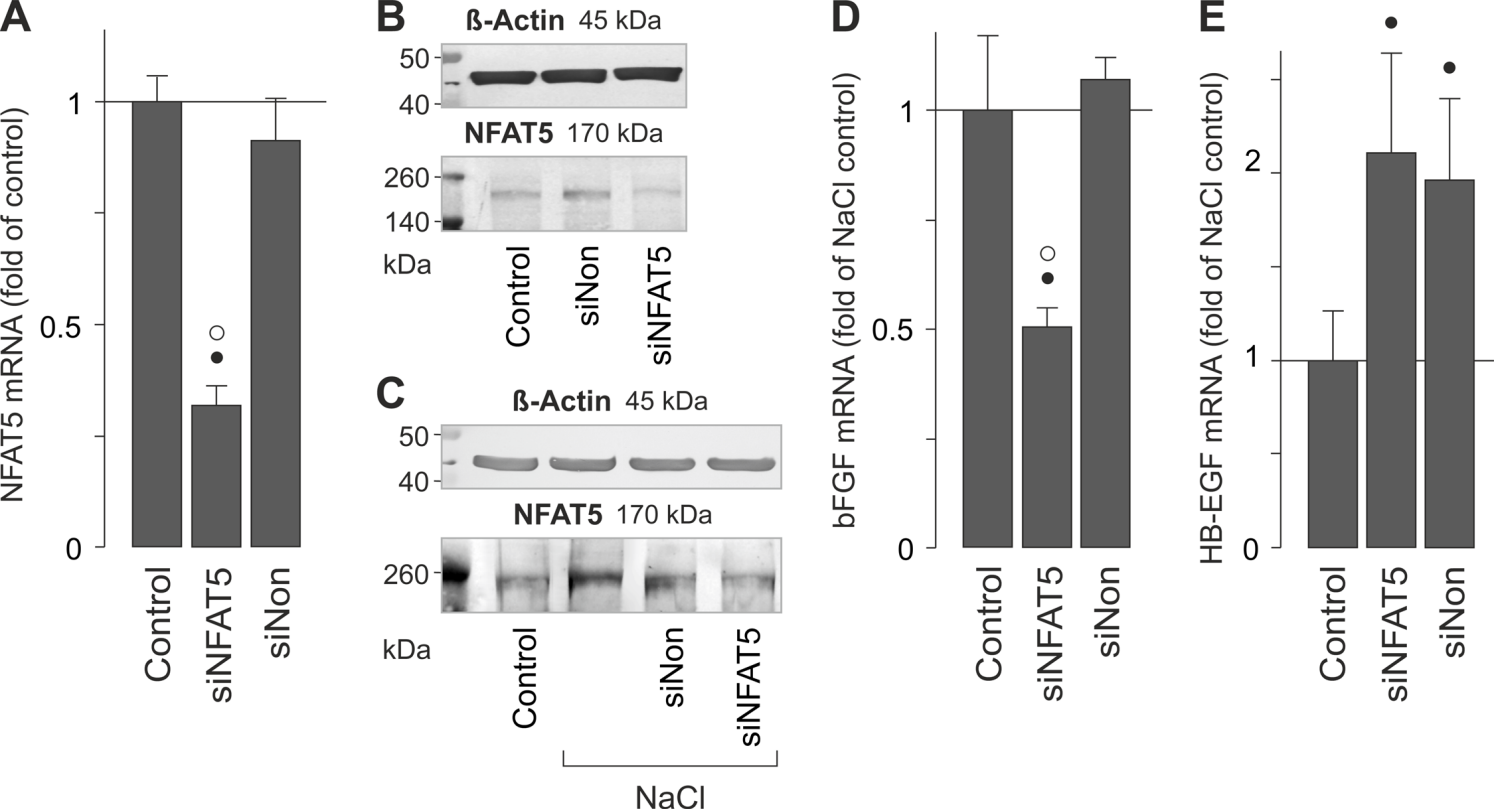
NFAT5 activity contributes to the hyperosmotic expression of the bFGF gene, but not of the HB-EGF gene. mRNA levels in cells cultured in hyperosmotic (+ 100 mM NaCl) medium were measured with real-time RT-PCR analysis (**A,D,E**). The cellular protein levels (**B,C**) were determined by Western blot analysis. After transfection of the cells with NFAT5 siRNA (siNFAT5) or nontargeted siRNA (siNon; 50 nM each) for 24 h, the cells were cultured 16 h in serum-free isoosmotic control medium. Thereafter, serum-free hyperosmotic medium was added for 6 h. (**A**). Transfection with siNFAT5 resulted in a reduction of the NFAT5 mRNA level under hyperosmotic conditions. (**B**). Effect of siRNA transfection on the cellular level of NFAT5 protein under isoosmotic control conditions. (**C**). Effect of siRNA transfection on the cellular level of NFAT5 protein under hyperosmotic conditions. (**D**). Transfection with siNFAT5 reduced the level of bFGF mRNA in cells cultured in hyperosmotic medium compared to nontransfected cells. Nontargeted siRNA was without effect. (**E**). Effects of siNFAT5 and nontargeted siRNA on the level of HB-EGF mRNA in cells cultured in hyperosmotic medium. Bars are means ± SEM of 3–7 independent experiments using cells from different donors. Significant difference *vs*. unstimulated control: *P<0.05. Significant difference *vs*. NaCl control: •*P*<0.05. Significant difference *vs*. nontargeted siRNA: ° *P*<0.05. In **B** and **C**, 35 (**B**) and 60 μg (**C**) of total protein were used for separation. β-Actin was used as a control for equal protein loading. Similar results were obtained in 3 independent experiments using cells from different donors.

In various cell systems, cellular survival under hyperosmotic conditions is dependent on the transcriptional activity of NFAT5 [40–42]. We described recently that extracellular hyperosmolarity increases the NFAT5 gene and protein expression, and induces DNA binding of NFAT5, in RPE cells [34]. In order to determine whether the hyperosmotic induction of bFGF and HB-EGF gene expression (Fig 1A) is dependent on the activity of NFAT5, we used siRNA to knock down NFAT5. NFAT5 siRNA reduced the level of NFAT5 transcripts by 50–70% in cells cultured in iso- (not shown) [34] and hyperosmotic media (Fig 4A), respectively. NFAT5 siRNA also reduced the level of NFAT5 protein in cells cultured in isoosmotic control medium before any treatment (Fig 4B) and in hyperosmotic medium (Fig 4C), respectively, while a nontargeted siRNA had no effect. Transfection with NFAT5 siRNA significantly (*P*<0.05) reduced the level of bFGF transcripts in cells stimulated with hyperosmotic medium (Fig 4D). A nontargeted siRNA did not alter the level of bFGF transcripts (Fig 4D). On the other hand, the siRNAs used induced slight increases in the level of HB-EGF mRNA in cells stimulated with high extracellular NaCl (Fig 4E), suggesting that NFAT5 activity is likely not involved in mediating the hyperosmotic expression of the HB-EGF gene. The data suggest that the bFGF gene, but not the HB-EGF gene, is (at least in part) transcriptionally activated by NFAT5 under hyperosmotic conditions in RPE cells.

### Hyperosmotic secretion of bFGF

**Fig 5:**
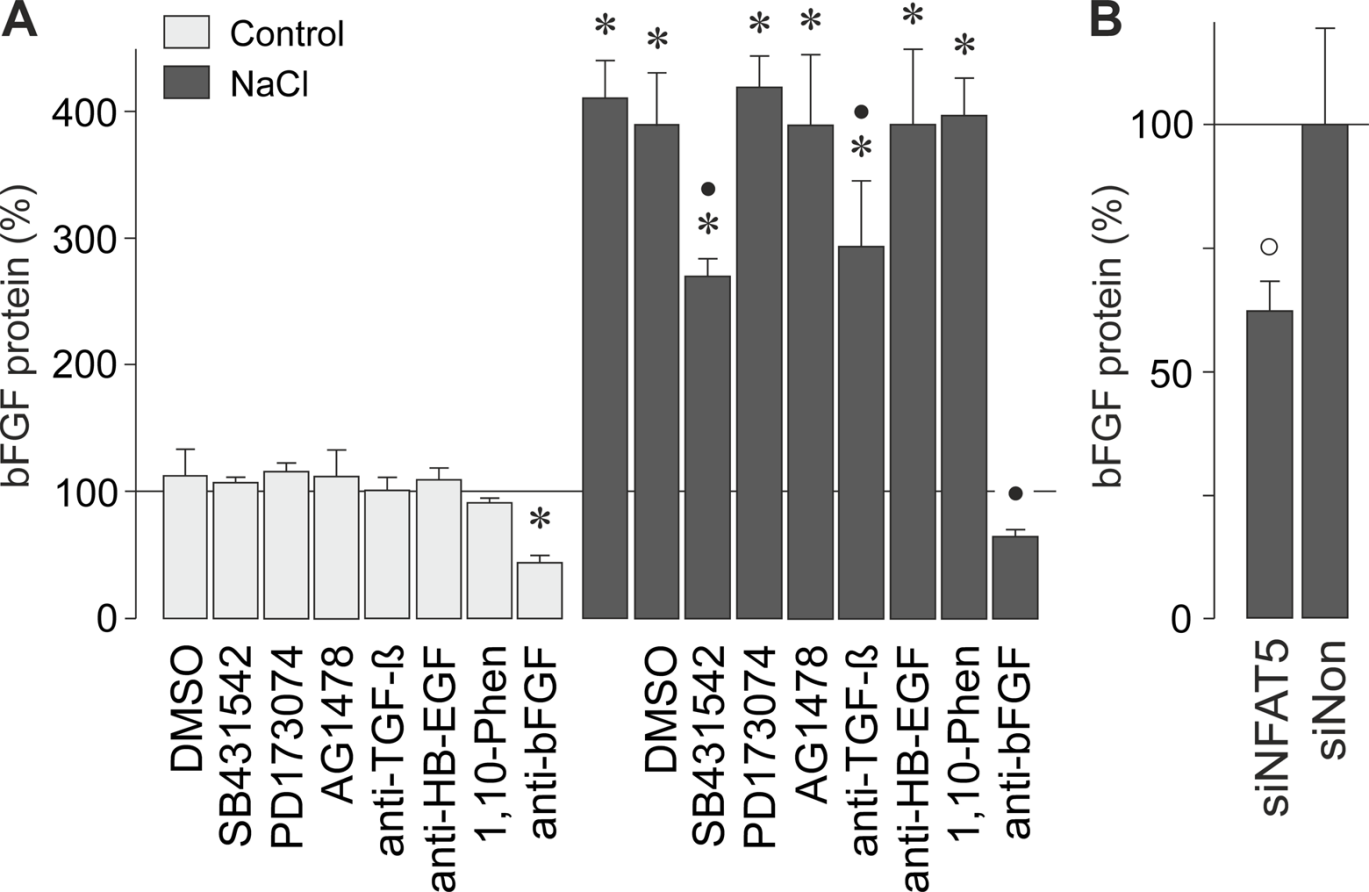
Hyperosmolarity induces secretion of bFGF that is in part dependent on autocrine/paracrine TGF-β signaling and NFAT5 activity. The level of bFGF protein was determined with ELISA in the media of cells cultured 6 h under iso- (control) and hyperosmotic (+ 100 mM NaCl) conditions. (**A**). The following pharmacological inhibitors were tested: the inhibitor of TGF-β1 superfamily activin receptor-like kinase receptors, SB431542 (10 μM), the FGF receptor kinase inhibitor, PD173074 (500 nM), the inhibitor of the EGF receptor tyrosine kinase, AG1478 (600 nM), neutralizing antibodies against TGF-β (anti-TGF-β) and HB-EGF (anti-HB-EGF), respectively (each at 20 μg/ml), as well as the broad-spectrum metalloproteinase inhibitor 1,10-phenanthroline (1,10-Phen; 10 μM). As control, a neutralizing antibody against bFGF (anti-bFGF; 20 μg/ml) was used. The vehicle control was made with dimethylsulfoxide (DMSO; 1:1000). (**B**). The hyperosmotic secretion of bFGF from cells transfected with NFTA5 siRNA (siNFAT5; 10 nM) was smaller than secretion of bFGF from cells transfected with nontargeted siRNA (siNon; 10 nM). Means ± SEM of 3–7 independent experiments using cells from different donors. Significant difference *vs*. unstimulated control: *P<0.05. Significant difference *vs*. NaCl control: •*P*<0.05. Significant difference *vs*. nontargeted siRNA: °*P*<0.05.

We found that autocrine/paracrine FGF signaling is involved in hyperosmotic induction of the bFGF and HB-EGF gene transcription (Fig 3A and 3B). In order to determine whether hyperosmolarity induces a release of bFGF protein, we measured the bFGF level in the cultured media with ELISA. Hyperosmotic challenge induced secretion of bFGF protein from RPE cells (Fig 5A). The hyperosmotic secretion of bFGF was significantly (*P*<0.05) reduced by the inhibitor of TGF-β1 superfamily activin receptor-like kinase receptors, SB431542, and a neutralizing anti-TGF-β antibody (Fig 5A). The hyperosmotic secretion of bFGF was not inhibited by the FGF receptor kinase inhibitor PD173074, the inhibitor of the EGF receptor tyrosine kinase AG1478, a neutralizing antibody against HB-EGF, and the broad-spectrum metalloproteinase inhibitor 1,10-phenanthroline (Fig 5A). The significant (*P*<0.05) decreases in the bFGF content of the media of cells cultured under iso- or hyperosmotic conditions in the presence of a neutralizing anti-bFGF antibody (Fig 5A) suggest that the cells secrete bFGF under both isoosmotic control and hyperosmotic conditions. The bFGF protein level was significantly (*P*<0.05) smaller in cultured media of cells transfected with NFAT5 siRNA compared to cells transfected with a nontargeted siRNA when the cells were stimulated with high NaCl (Fig 5B). The data suggest that a part of bFGF released from the cells under hyperosmotic stress was derived from newly synthesized bFGF protein. We were unable to detect soluble HB-EGF with ELISA in the cultured media of cells that were grown 6 h under iso- or hyperosmotic (+ 100 mM NaCl) conditions (not shown).

### Cell viability

**Fig 6:**
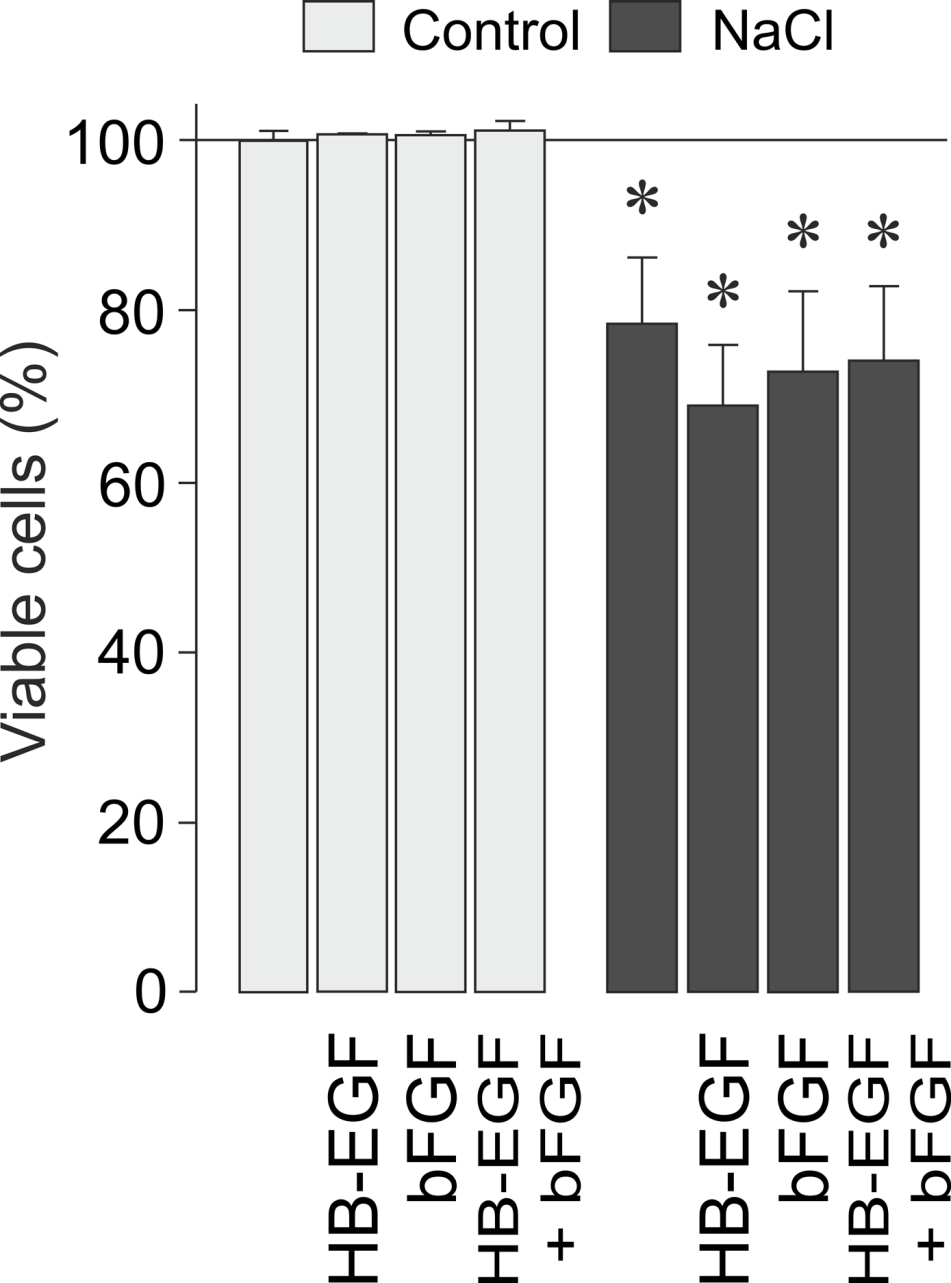
HB-EGF and bFGF do not alter the viability of RPE cells. Both factors were tested at 10 ng/ml. The data were obtained in cells cultured 24 h in iso- (control) and hyperosmotic (+ 100 mM NaCl) media, respectively. Data are means ± SEM of 3–6 independent experiments using cells from different donors. Significant difference *vs*. isoosmotic control: *P<0.05.

bFGF and HB-EGF are protective factors that increase the survival of photoreceptors, retinal neurons, and RPE cells [22,43–45]. In order to determine whether growth factor signaling may enhance the survival of RPE cells, we tested exogenous bFGF and HB-EGF under iso- and hyperosmotic conditions. As shown in Fig 6, the viability of RPE cells was significantly (*P*<0.05) reduced under hyperosmotic conditions compared to isoosmotic control. The hyperosmotic decrease of the cell viability was not prevented by exogenous bFGF and HB-EGF (Fig 6).

### Effects of vegetable polyphenols on the gene expression of bFGF, HB-EGF, and NFAT5

**Fig 7:**
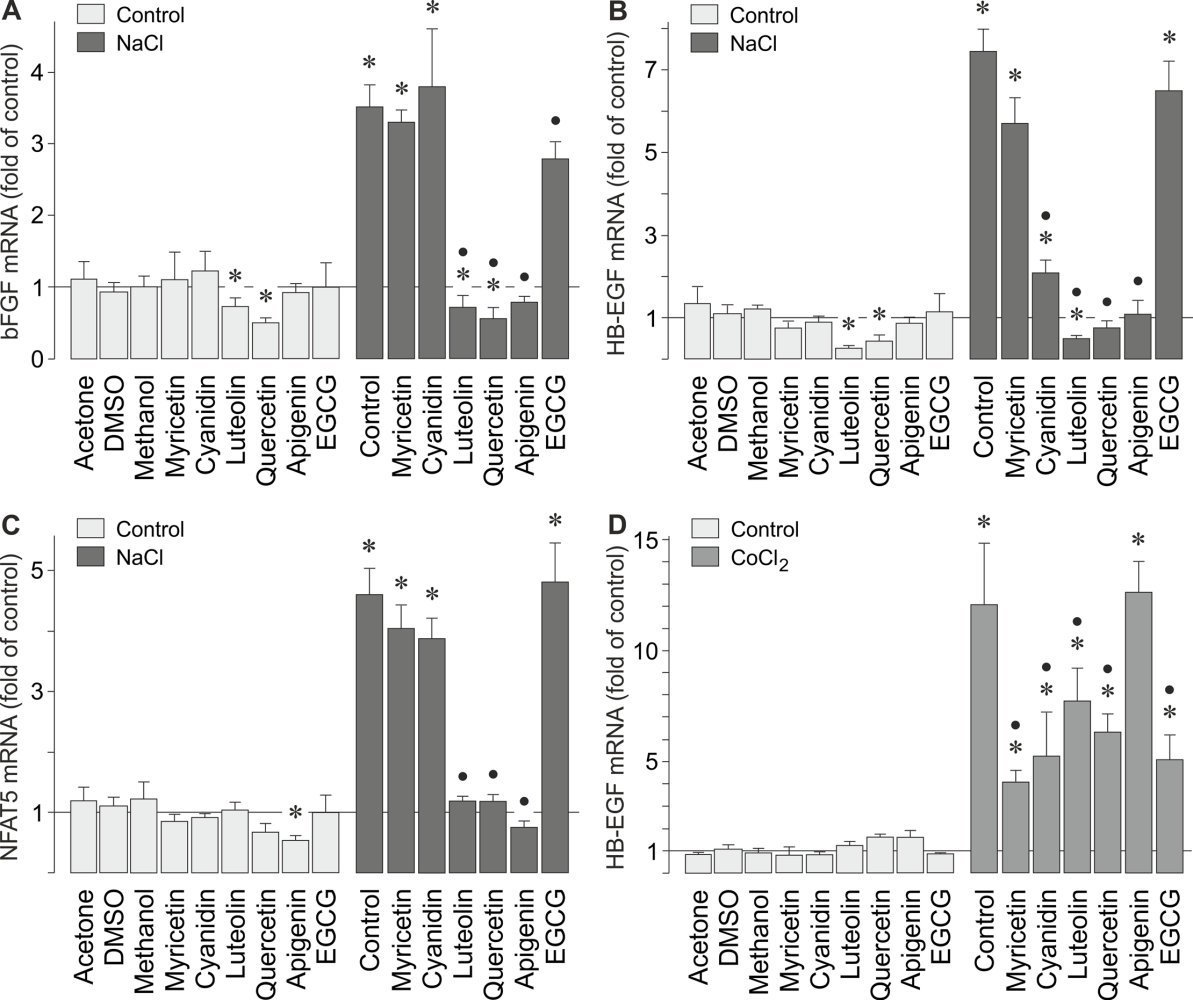
Distinct vegetable polyphenols inhibit the hyperosmotic expression of bFGF, HB-EGF, and NFAT5 genes. bFGF (**A**), HB-EGF (**B,D**), and NFAT5 (**C**) mRNA levels in cells cultured 6 (**A-C**) and 24 h (**D**) under hyperosmotic (**A-C**) and hypoxic conditions (**D**) were determined with real-time RT-PCR analysis. Hyperosmolarity was induced by addition of NaCl (100 mM) to the culture medium. Chemical hypoxia was induced by addition of CoCl_2_ (150 μM). The following polyphenols were tested: myricetin (50 μM), cyanidin (100 μM), luteolin (50 μM), quercetin (100 μM), apigenin (50 μM), and EGCG (50 μM). Vehicle controls were made with acetone (0.1%), dimethylsulfoxide (DMSO; 0.1%), and methanol (0.2%). Means ± SEM of 3–6 independent experiments using cells from different donors. Significant difference *vs*. unstimulated control: *P<0.05. Significant difference *vs*. NaCl control: •*P*<0.05.

Oxidative stress induced HB-EGF gene expression in RPE cells (Fig 1F). Vegetable polyphenols are suggested to have beneficial effects in retinal diseases [46–48] mainly by their antioxidant effects [49,50]. Therefore, we determined whether the hyperosmotic expression of the bFGF and HB-EGF genes is inhibited by vegetable polyphenols. We tested the most abundant catechin of green tea, (-)-epigallocatechin-3-gallate (EGCG), apigenin from celery and parsley, myricetin from black tea, grapes, and walnuts, cyanidin from various plants such as red cabbage, blueberries, and strawberries, luteolin from parsley, and quercetin from bulbs. The polyphenols at the concentrations used in the present study were recently described to affect physiological properties of RPE cells including the production of VEGF [51]. The hyperosmotic expression of the bFGF, HB-EGF, and NFAT5 genes was fully prevented by luteolin, quercetin, and apigenin (Fig 7A–7C). Cyanidin inhibited the hyperosmotic expression of the HB-EGF gene (Fig 7B), but not of the bFGF (Fig 7A) and NFAT5 genes (Fig 7C). The hypoxic expression of the HB-EGF gene was decreased by all polyphenols investigated with the exception of apigenin (Fig 7D).

## Discussion

The synergistic action of various angiogenic factors is suggested to be critical for the development of choroidal neovascularization [11,12], a characteristic of wet AMD [2]. Systemic hypertension is a risk factor of wet AMD [23–25]. The major condition that causes acute hypertension is the increase of the extracellular osmolarity following high intake of dietary salt [26,27]. Therefore, we investigated whether high extracellular osmolarity alters the expression of angiogenic factors in RPE cells. We found recently that extracellular hyperosmolarity induces the production of VEGF in RPE cells [34]. In the present study, we describe that extracellular hyperosmolarity also induces the expression of bFGF and HB-EGF genes in RPE cells while the gene expression of further cytokines was moderately or not altered (Fig 1A–1C). We found that high NaCl-induced hyperosmolarity, CoCl_2_-induced hypoxia, and H_2_O_2_-induced oxidative stress regulate differentially the gene expression of the cytokines. While the expression of the HB-EGF gene increased in response to hyperosmolarity (Fig 1A), hypoxia (Fig 1E), and oxidative stress (Fig 1F), the expression of the bFGF gene was increased under hyperosmotic conditions (Fig 1A) and remained unaltered under hypoxic and oxidative stress conditions (Fig 1E and 1F). It has been shown previously that hypoxia induces the secretion of VEGF, but not of bFGF, from RPE cells [38]. The present data are also in agreement with previous studies using different cell systems which showed that extracellular hyperosmolarity induces the expression of HB-EGF [52,53]. The increase in the level of VEGF mRNA and the delayed decrease in the level of PEDF mRNA induced by chemical hypoxia (Fig 1E) are consistent with previous studies which showed that hypoxia induces upregulation of VEGF and downregulation of PEDF in the retina [4,54].

It has been shown recently that hyperosmotic stress induces phosphorylation of various key intracellular signal transduction molecules in RPE cells including p38 MAPK and ERK1/2 [34]. In various cell systems, extracellular hyperosmolarity also induces activation of JNK and PI3K signal transduction pathways and, in addition to p38α MAPK and ERK1/2 activities, JNK and PI3K activities may be involved in mediating the stimulatory effect of extracellular hyperosmolarity on the gene expression [42,55–60]. We found differences in the involvement of intracellular signal transduction pathways in the hyperosmotic induction of bFGF and HB-EGF gene transcription. The hyperosmotic expression of the bFGF gene is, at least in part, mediated by activation of p38α/β MAPK, JNK, and PI3K signal transduction pathways, but not by activation of ERK1/2 (Fig 2A). The hyperosmotic expression of the HB-EGF gene is dependent on activation of p38α/β MAPK, ERK1/2, and JNK signal transduction pathways, but not on the activation of PI3K (Fig 2B). On the other hand, the hypoxia-induced expression of the HB-EGF gene was independent on the activation of the signal transduction pathways (Fig 2C). The data suggest that extracellular hyperosmolarity induces activation of various intracellular signal transduction pathways that differentially regulate the gene expression of angiogenic factors in RPE cells.

The hyperosmotic expression of the bFGF and HB-EGF genes is in part induced by autocrine growth factor signaling. Hyperosmotic stress induced secretion of bFGF (Fig 5A) but not of HB-EGF (not shown). It has been shown previously in other cell systems that hyperosmotic stress induces a matrix metalloproteinase activity leading to cell surface cleavage of pro-HB-EGF and subsequent EGF receptor activation [61]. However, EGF receptor agonists are unlikely to mediate the hyperosmotic induction of the bFGF and HB-EGF gene expression in RPE cells because the inhibitor of the EGF receptor tyrosine kinase, AG1478, had no effects (Fig 3A and 3B). In addition, the metalloproteinase inhibitor 1,10-phenanthroline did not prevent the hyperosmotic expression of the bFGF and HB-EGF genes (Fig 3A and 3B). Autocrine PDGF and VEGF signaling is also unlikely to mediate the hyperosmotic induction of HB-EGF gene transcription because both factors were shown to have no effects on the HB-EGF gene expression in RPE cells [14]. We found that the hyperosmotic expression of the bFGF and HB-EGF genes was decreased by inhibitors of TGF-β1 superfamily activin receptor-like kinase receptors and the FGF receptor kinase (Fig 3A and 3B). The data suggest that the hyperosmotic expression of bFGF and HB-EGF genes is in part mediated by a release of TGF-ß and FGF from the cells and autocrine/paracrine activation of the respective receptors. This assumption is in agreement with a previous study which showed that TGF-ß and bFGF are inducers of HB-EGF gene expression in RPE cells [14]. On the other hand, the hypoxic expression of the HB-EGF gene was independent on activation of the receptors investigated (Fig 3C). In addition, we found evidence that the hyperosmotic induction of the VEGF gene transcription is mediated by autocrine/paracrine TGF-β1 and FGF signaling (Fig 3D). TGF-β is a well-known inducer of VEGF in RPE cells [62], and bFGF was shown to induce the production of VEGF in retinal glial cells [18,19].

Hyperosmotic stress induced secretion of bFGF from RPE cells (Fig 5A). The hyperosmotic secretion of bFGF was partially prevented by an inhibitor of TGF-β1 superfamily activin receptor-like kinase receptors and a neutralizing anti-TGF-β antibody, respectively (Fig 5A). This suggests that, in addition to further signaling mechanisms, autocrine/paracrine TGF-β signaling is involved in mediating the hyperosmotic secretion of bFGF. It is unlikely that autocrine/paracrine EGF receptor signaling mediated, for example, by HB-EGF is involved in mediating the hyperosmotic secretion of bFGF, for the following reasons: the inhibitor of the EGF receptor tyrosine kinase, AG1478, did not decrease the hyperosmotic secretion of bFGF (Fig 5A); a neutralizing antibody against HB-EGF had no effect (Fig 5A); the broad-spectrum metalloproteinase inhibitor 1,10-phenanthroline was without effect (Fig 5A); and we were unable to detect soluble HB-EGF with ELISA in the cultured media of the cells (not shown). Apparently, hyperosmolarity induces expression of the HB-EGF gene (Fig 1A–1C) but not shedding of soluble HB-EGF protein from the membrane-bound precursor. The failure of the FGF receptor kinase inhibitor PD173074 in inhibiting the hyperosmotic secretion of bFGF (Fig 5A) suggests that hyperosmolarity-induced autocrine/paracrine FGF signaling stimulates the bFGF gene expression (Fig 3A) but not the secretion of bFGF.

We found evidence that the hyperosmotic transcription of the bFGF gene (Fig 4D), but not of the HB-EGF gene (Fig 4E), was (at least in part) induced by the activity of NFAT5. However, the present results do not exclude the possibility that additional transcription factors contribute to the transcriptional activation of the bFGF gene under osmotic stress conditions. We showed recently that extracellular hyperosmolarity, but not hypoxia, induces increases of the NFAT5 mRNA and protein expression, and DNA binding of NFAT5, in RPE cells [34]. It remains to be determined whether the activation of intracellular signal transduction pathways (Fig 2A) contributes to the activation of NFAT5 under hyperosmotic conditions in RPE cells. The inhibitory effect of NFAT5 siRNA on the hyperosmotic secretion of bFGF (Fig 5B) may suggest that a part of bFGF secreted from the cells is newly produced during the hyperosmotic stimulation.

It has been shown in various cell systems that the release of bFGF and the subsequent activation of the FGF receptor kinase increase the cellular survival under conditions of altered extracellular osmolarity [61,63]. bFGF and HB-EGF are known survival factors in the retina, e.g., of photoreceptors, retinal neurons, and RPE cells [22,43–45]. The viability of RPE cells is decreased under hyperosmotic conditions (Fig 6). However, exogenous bFGF and HB-EGF did not increase the cell viability under iso- and hyperosmotic conditions (Fig 6), suggesting that these factors are not important for the survival of RPE cells under the conditions tested. The reason for the present findings, which are different from studies in other cell systems [61,63], is unclear and may be related to the use of various cell systems and different stimulation conditions (hypoosmotic vs. hyperosmotic stimulation and use of high NaCl or sorbitol to increase the extracellular osmolarity).

Oxidative stress is an important pathogenic factor of AMD [64]. High extracellular NaCl is known to cause oxidative stress which contributes to the activation of NFAT5 [35,36,55]. We tested whether the hyperosmotic expression of the bFGF and HB-EGF genes can be reduced by vegetable polyphenols. We found that various polyphenols (luteolin, quercetin, apigenin) abrogated the hyperosmotic expression of these genes (Fig 7A and 7B). It has been shown that high extracellular osmolarity, but not hypoxia, induces the expression of the NFAT5 gene in RPE cells [34]. We found that the above-mentioned polyphenols also abrogated the hyperosmotic expression of the NFAT5 gene (Fig 7C). However, it remains unclear whether the inhibitory effects of distinct polyphenols on the hyperosmotic expression of the bFGF gene (Fig 7A) are mediated by inhibiton of NFAT5 production or activity. NFAT5 activity is known to be independently regulated in a cell type-specific manner by different mechanisms at multiple levels including mRNA and protein expression, mRNA stability, nucleocytoplasmic shuttling, DNA binding, and transcriptional and transactivational activities [55,65]. It has been shown that distinct antioxidants inhibit the high NaCl-induced NFAT5 transcriptional and transactivational activities [55,64]. However, the inhibitory effects of distinct polyphenols on the hyperosmotic expression of the bFGF gene (Fig 7A) may also result from polyphenol-induced inhibition of intracellular signal transduction pathways such as ERK1/2 and PI3K-Akt pathways [51,55,66]. The molecular mechanisms of the polyphenol action on the hyperosmotic gene expression in RPE cells remain to be determined in future experiments. The hypoxic gene expression of HB-EGF was decreased, but not abrogated, by various polyphenols (Fig 7D), suggesting that the hypoxic and hyperosmotic expression of the HB-EGF gene is differently regulated by intracellular signal transduction pathways.

We found significant effects of high salt on the expression of bFGF and HB-EGF genes when more than 10 mM NaCl were added to the culture medium (Fig 1B), i.e., when the extracellular osmolarity was increased above ~307 mosm/kg H_2_O from a control osmolarity of 287.5 ± 1.6 mosm/kg H_2_O. An increase of the extracellular NaCl concentration by 30 mM (i.e., to an extracellular osmolarity of 346.9 ± 2.3 mosm/kg H_2_O) induced significant expression of bFGF and HB-EGF genes in RPE cells (Fig 1B). It is generally accepted that the highest pathological blood osmolarity in human subjects is around 360 mosm/kg H_2_O [67,68]. Because the basolateral membranes of RPE cells *in situ* have contact to the blood of the fenestrated choroidal vessels, the present results may have relevance for *in-vivo* conditions. Because the plasma osmolarity and the salt sensitivity of the blood pressure increase with age [28,69], high dietary salt may have effects in particular in aged salt-sensitive individuals.

Systemic hypertension is a risk factor of wet AMD [23–25]; however, antihypertensive medications do not decrease the risk of the disorder [29]. The present results support the assumption that high intake of dietary salt resulting in raised extracellular osmolarity [26,27] may have direct effects on RPE cells, independent from hypertension. These effects include the stimulation of the gene expression of various angiogenic factors including bFGF and HB-EGF, and the expression of IL-1β (Fig 1A). The salt-induced production of angiogenic factors may stimulate the development of choroidal neovascularization and edema, while IL-1β may aggravate retinal inflammation associated with AMD. However, whether these effects aggravate the pathogenesis of AMD *in vivo*, and whether low salt diet and/or increased water intake (that reduces the blood osmolarity) have greater protective effects than antihypertensive medications, remain to be established.
